# Distinction Between Variability-Based Modulation and Mean-Based Activation Revealed by BOLD-fMRI and Eyes-Open/Eyes-Closed Contrast

**DOI:** 10.3389/fnins.2018.00516

**Published:** 2018-07-31

**Authors:** Pei-Wen Zhang, Xiu-Juan Qu, Shu-Fang Qian, Xin-Bo Wang, Rui-Di Wang, Qiu-Yue Li, Shi-Yu Liu, Lihong Chen, Dong-Qiang Liu

**Affiliations:** Research Center of Brain and Cognitive Neuroscience, Liaoning Normal University, Dalian, China

**Keywords:** BOLD, dynamics, variability, mean, eyes-open/eyes-closed

## Abstract

Recent BOLD-fMRI studies have revealed spatial distinction between variability- and mean-based between-condition differences, suggesting that BOLD variability could offer complementary and even orthogonal views of brain function with traditional activation. However, these findings were mainly observed in block-designed fMRI studies. As block design may not be appreciate for characterizing the low-frequency dynamics of BOLD signal, the evidences suggesting the distinction between BOLD variability and mean are less convincing. Based on the high reproducibility of signal variability modulation between continuous eyes-open (EO) and eyes-closed (EC) states, here we employed EO/EC paradigm and BOLD-fMRI to compare variability- and mean-based EO/EC differences while the subjects were in light. The comparisons were made both on block-designed and continuous EO/EC data. Our results demonstrated that the spatial patterns of variability- and mean-based EO/EC differences were largely distinct with each other, both for block-designed and continuous data. For continuous data, increases of BOLD variability were found in secondary visual cortex and decreases were mainly in primary auditory cortex, primary sensorimotor cortex and medial nuclei of thalamus, whereas no significant mean-based differences were observed. For the block-designed data, the pattern of increased variability resembled that of continuous data and the negative regions were restricted to medial thalamus and a few clusters in auditory and sensorimotor networks, whereas activation regions were mainly located in primary visual cortex and lateral nuclei of thalamus. Furthermore, with the expanding window analyses we found variability results of continuous data exhibited a rather slower dynamical process than typically considered for task activation, suggesting block design is less optimal than continuous design in characterizing BOLD variability. In sum, we provided more solid evidences that variability-based modulation could represent orthogonal views of brain function with traditional mean-based activation.

## Introduction

In traditional task-based functional magnetic resonance imaging (fMRI) studies, activation regions were typically identified by comparing the temporal mean of a task related time course with baseline. Signal variability was usually conceived as uninformative noise. However, recent evidences have suggested that fMRI signal variability may also be functionally relevant (See reviews in Garrett et al., 2013b; Grady and Garrett, 2014). For example, blood oxygenation level dependent (BOLD) variability could predict age (Garrett et al., 2010, 2011, 2013a) and task performances (Garrett et al., 2011, 2013a; Wutte et al., 2011; Protzner et al., 2013), mediate the relationship between them (Samanez-Larkin et al., 2010; Baum and Beauchamp, 2014) and respond to different task conditions (Duff et al., 2008; Ricciardi et al., 2013; Garrett et al., 2015; Guitart-Masip et al., 2015). Of particular interest, a few studies have revealed that the between-condition differences in signal variability have distinct spatial patterns from the traditional activation regions (Protzner et al., 2013; Garrett et al., 2014; Guitart-Masip et al., 2015). These findings implied that traditional activation could not represent a complete description of human brain response, and brain variability may provide complementary and even orthogonal views (Garrett et al., 2013b).

So far, the distinction between variability- and mean-based measurements was mainly observed in block-designed fMRI studies (e.g., Garrett et al., 2011, 2014; Protzner et al., 2013; Guitart-Masip et al., 2015), but it remains uncertain whether block design is appropriate for examination of BOLD variability. The discrete and short blocks could probably disrupt the continuous and low-frequency fluctuations (Biswal et al., 1995; Lowe et al., 1998; Cordes et al., 2000, 2001; Whitlow et al., 2011; Birn et al., 2013; Gonzalez-Castillo et al., 2014; Tomasi D. G. et al., 2016) and thus the block-concatenated data cannot ensure accurate measurement of brain variability. If so, the evidences that distinction between variability and traditional activation would be less convincing. By contrast, continuous design may be more suitable for the slow fluctuation. The idea of continuous design is from the field of resting state fMRI (rs-fMRI), which means that data of only one condition is scanned within the whole fMRI run. In these studies, researchers are often interested in the regional fluctuation rather than mean level of fMRI signal (Fransson, 2006; Dong et al., 2012; Zhang and Zang, 2015).

Using continuous design, previous studies have revealed that non-visual sensory modalities, including bilateral primary auditory cortex (PAC) and primary sensorimotor cortex (PSMC), showed significantly decreased signal power (or variability) between continuous eyes-open (EO) and eyes-closed (EC) states. Such findings are highly consistent across different groups (McAvoy et al., 2008; Yan et al., 2009; Jao et al., 2013; Liu et al., 2013; Yuan et al., 2014). These seem to support the spatial distinction between variability- and mean-based activities. However, widespread deactivation (i.e., decrease of signal mean) of PSMC has actually also been observed in block-designed EO/EC paradigm, though in darkness (Marx et al., 2003). For EO/EC paradigm, it is somewhat surprising that no studies have directly compared between BOLD derived variability- and mean-based between-condition differences under the same level of room illuminance. Although Zou et al. (2015) have found BOLD power differences showed little overlap with cerebral blood flow (CBF)-based mean differences between EO and EC, their results are confounded by different sensitivity of acquisition techniques, i.e., BOLD vs. arterial spin labeling that measures CBF (Tjandra et al., 2005; Federspiel et al., 2006). Though they also made comparison of CBF-based power with mean CBF, the low temporal resolution (an effective TR of 9 s) of CBF time series precludes it from accurately measuring signal dynamics.

In this study, we compared the spatial distribution between variability- and mean-based EO/EC differences revealed by BOLD-fMRI, within the same subjects while keeping the scanner room lightened. In order to perform comprehensive comparisons, signal mean and variability were compared both for block-designed and continuous data. Additionally, to evaluate whether block design is suitable for study of BOLD variability, we used the expanding window approach (Shirer et al., 2012; Birn et al., 2013; Zuo et al., 2013; Tomasi D. G. et al., 2016) to examine the temporal evolution of variability-based EO/EC differences on the continuous data.

## Materials and methods

### Participants

Thirty-six healthy participants (21.5 ± 2.27 years, 18 females) were recruited. Each provided written informed consent and was screened with a questionnaire to ensure no history of brain injury, neurological illness or psychiatric disorders. This study was approved by the ethics committee of Institutional Review Board of the Research Center of Brain and Cognitive Neuroscience, Liaoning Normal University.

### Experimental procedures

There were totally four sessions for each participant, each lasting 8 min. Specifically, an EC resting state session was firstly acquired (the data was not analyzed in this study). Then, two continuous resting state sessions, EO and EC, were acquired and counter-balanced across participants. In these sessions, the participants were instructed to lie in the scanner quietly with their eyes open or closed, not to fall asleep, to be as motionless as possible, and not to think about anything in particular. Finally, the participants underwent an EO/EC block-designed session, in which subjects were instructed to alternately open and close their eyes for 30 s in respond to an acoustic signal via earphones. This session contained 8 EO and 8 EC blocks. A black and blank screen without fixation was always presented. All participants reported that they had not fallen asleep during the scanning.

### Data acquisition

MRI data was acquired using a GE Discovery MR750 3-Tesla scanner at the Research Center of Brain and Cognitive Neuroscience, Liaoning Normal University. The functional images were obtained by using an echo-planar imaging (EPI) sequence with the following parameters: 33 axial slices, slice thickness/gap = 3.5/0.7 mm, flip angle = 90°, TR = 2,000 ms, TE = 30 ms, in-plane resolution = 64 × 64, field of view (FOV) = 224 × 224 mm^2^. For the purpose of spatial normalization, we acquired a 3D T1-weighted image for each subject using a spoiled gradient-recalled pulse sequence (192 sagittal slices, slice thickness = 1.0 mm, flip angle = 12°, TR = 6,652 ms, TE = 2.93 ms, inversion time (TI) = 450 ms, in-plane resolution = 256 × 256, FOV = 256 × 256 mm^2^).

### Data preprocessing

The EPI data were preprocessed by using the toolbox for Data Processing & Analysis for Brain Imaging (DPABI, http://rfmri.org/DPABI, Yan et al., 2016). Preprocessing steps included: (i) removal of the first 10 volumes of functional images, (ii) slice-timing correction, (iii) head motion correction, (iv) spatial normalization to Montreal Neurological Institute (MNI) space with a resampling resolution of 3 × 3 × 3 mm3, (v) spatial smoothing with a 4-mm Gaussian kernel along all three directions, (vi) scaling image intensity to a grand session mean of 1000. Notably, two participants were excluded due to excessive head motion (more than 1.5 mm of maximal translation in any direction of x, y, or z or 1.5° of any angular motion throughout the course of scan).

The Friston 24-parameter model (6 motion parameters, 6 motion parameters one time point before, and 12 corresponding squared items, Friston et al., 1996; Yan et al., 2013) was utilized to regress out head motion effects in this study. The averaged signals from white matter (WM) and cerebral spinal fluid (CSF) and the low-frequency drifts (i.e., linear, quadratic and cubic trends) were also regressed out.

### Signal mean-based EO/EC differences

For the block-designed data, we carried out a standard general linear model (GLM) analysis to reveal differences in mean signal level between EO and EC (i.e., EO/EC activation) by using the SPM12 software (https://www.fil.ion.ucl.ac.uk/spm). For each individual, task regressor representing alternated EO and EC blocks was generated by convolving a boxcar function with the canonical hemodynamic response function as well as its time and dispersion derivatives. Group-level statistical analysis was performed on the individual-level beta images by using permutation test implemented by PALM (1,000 permutations) (Winkler et al., 2016). Multiple comparisons were adjusted for using threshold-free cluster enhancement (TFCE, Smith and Nichols, 2009). The corrected P values were thresholded at *p* < 0.05.

For the purpose of comprehensive comparisons, we also examined the EO/EC differences in signal mean on the continuous data. Signal mean was calculated for each voxel and condition (i.e., EO and EC), respectively. And then individual signal-mean maps were entered into group-level statistical analysis. The statistical and multiple comparison correction procedures were the same as described above. Of note, image intensity scaling during preprocessing may bias the differences in signal mean, in this section we also analyzed data without intensity scaling.

### Signal variability-based EO/EC differences

In this study, we used standard deviation (SD) to quantify the variability of BOLD signal. A variety of metrics have been utilized to characterize BOLD variability, such as power spectrum (Duff et al., 2008; McAvoy et al., 2008), variance (Jao et al., 2013) and amplitude of low-frequency fluctuation (ALFF, Zang et al., 2007). Mathematically, these metrics are almost equivalent to each other. Compared with the other metrics, however, SD has the same scale with the original time series. Moreover, it is more appropriate to measure the temporal variability of short time series, such as that of one block in block-designed data (as did in Garrett et al., 2010, 2011, 2013a, 2014; Protzner et al., 2013; Guitart-Masip et al., 2015).

SD was calculated for each voxel both on the block-designed and continuous data. For the block-designed data, time series was firstly concatenated across blocks belonging to the same condition in a similar way with the previous variability studies (Protzner et al., 2013; Garrett et al., 2014; Guitart-Masip et al., 2015). Before concatenation, signal mean was subtracted for each block. To minimize the effects of hemodynamic delay from previous conditions, the first four volumes (8 s) were removed and two volumes (4 s) of the next block were added for each block (Liang et al., 2015). Finally, we calculated SD for the EO and EC conditions, respectively. To control for the global effects, the individual SD maps (both for block-designed and continuous data) were divided by the global mean SD (Zang et al., 2007; Yan et al., 2013). However, considering that the validity of such manipulation has not been fully established (Yan et al., 2013), we also presented results without global mean normalization (GMN) (Please see the Supplementary Material).

Voxel-wise statistical analyses were carried out on the individual-level SD maps (for the concatenated block-designed and continuous data, respectively) to reveal the EO/EC differences. Multiple comparison correction was performed by using the same procedures as described before. The corrected *P*-values were thresholded at *p* < 0.05. Finally, the comparisons between mean- and SD-based results were made by visual inspection.

### Temporal evolution of SD-based EO/EC differences

To evaluate whether block deign is suited for BOLD variability analysis, we used expanding window approach (Shirer et al., 2012; Birn et al., 2013; Zuo et al., 2013; Tomasi D. G. et al., 2016) to examine the temporal evolution of SD-based EO/EC differences on the continuous data. In particular, we constructed a set of new datasets with increasing window lengths from the original data of full length (i.e., 460 s). The shortest time window had 15 time points (i.e., 30 s, to match the block length in block-designed session). Subsequently, the window was expanded 15 time points (30 s) in each step. In addition to the full-length data, a total of 15 constructed datasets with different window lengths were obtained. We then calculated SD maps for each window and condition. The resulting SD maps were entered into group-level statistical analyses to reveal the EO/EC differences for each window.

For the illustration purpose, we then calculated dice coefficient to evaluate the similarity between EO/EC difference maps based on each constructed dataset and the full-length dataset. Dice coefficient was calculated with the following equation:

$$\begin{array}{l} Dice=\frac{{\text{2}\times V}_{overlap}}{V_{full}+V_{recon}} \end{array}$$

where *V*_*full*_ and *V*_*recon*_ represent the number of supra-threshold voxels in the results of full-length dataset and a constructed dataset, respectively. *V*_*overlap*_ is the number of supra-threshold voxels in the intersection areas.

## Results

### Signal mean-based EO/EC differences

**Figure 1:**
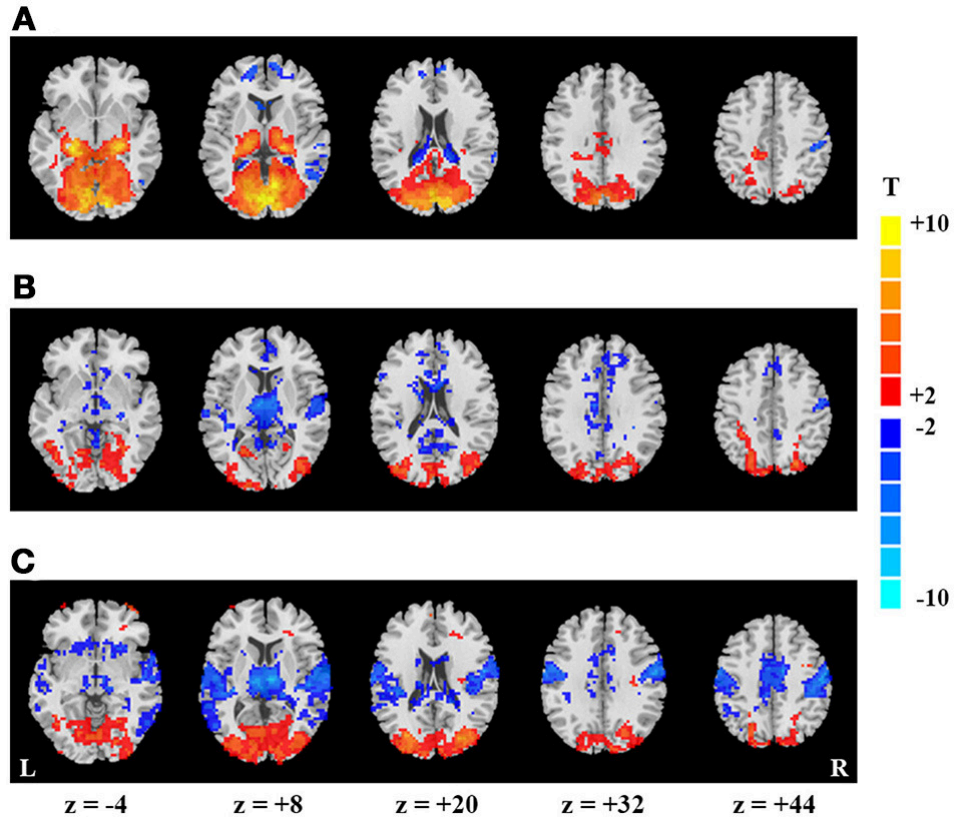
Spatial maps of mean- and SD-based EO/EC differences. Activation map **(A)**, and SD-based EO/EC differences for the blocked-designed **(B)** and continuous **(C)** data analyzed with GMN. The corrected *P*-values were thresholded at *p* < 0.05. The warm colors indicate the regions with significantly increased activities in EO than EC, and the cool colors indicate the opposite (L, left hemisphere; R, right hemisphere).

For the block-designed data, the activation regions were mainly located in PVC, lateral thalamus and a few voxels in right PSMC and PAC (*p* < 0.05, corrected, Figure 1A). For the continuous data, we did not found significant differences between EO and EC conditions both for the data with and without intensity scaling (*p* < 0.05, corrected).

### SD-based EO/EC differences

For the block-designed data, regions with significantly increased SD were observed in bilateral secondary visual cortex as well as one small cluster in PVC. Decreased regions were mainly in right PSMC, PAC, thalamus, some small clusters in middle cingulate cortex and superior medial frontal cortex (*p* < 0.05, corrected, Figure 1B). For the continuous data, both positive and negative regions were larger than in block design. Moreover, the decreased regions also exhibited symmetric distribution and included bilateral PAC, PSMC, supplementary motor area, middle cingulate cortex and medial thalamus (*p* < 0.05, corrected, Figure 1C).

As GMN may introduce artificial differences, we also presented the results without GMN. For the block-designed data, we only found the increased SD in visual cortex and decrease in thalamus (*p* < 0.05, corrected, Supplementary Figure 1B). The SD-based differences of continuous data showed similar patterns with those analyzed with GMN (*p* < 0.05, corrected, Supplementary Figure 1C). The differences between block-designed and continuous data were much more obvious in the data analyzed without GMN.

By visual inspection, SD and activation results were only overlapped in thalamus and a portion of visual cortex. Nevertheless, the significant changes were more prominent in PVC and lateral thalamus for activation, but were more significant in secondary visual cortex and medial thalamus for SD results.

### Temporal evolution of SD-based differences

**Figure 2:**
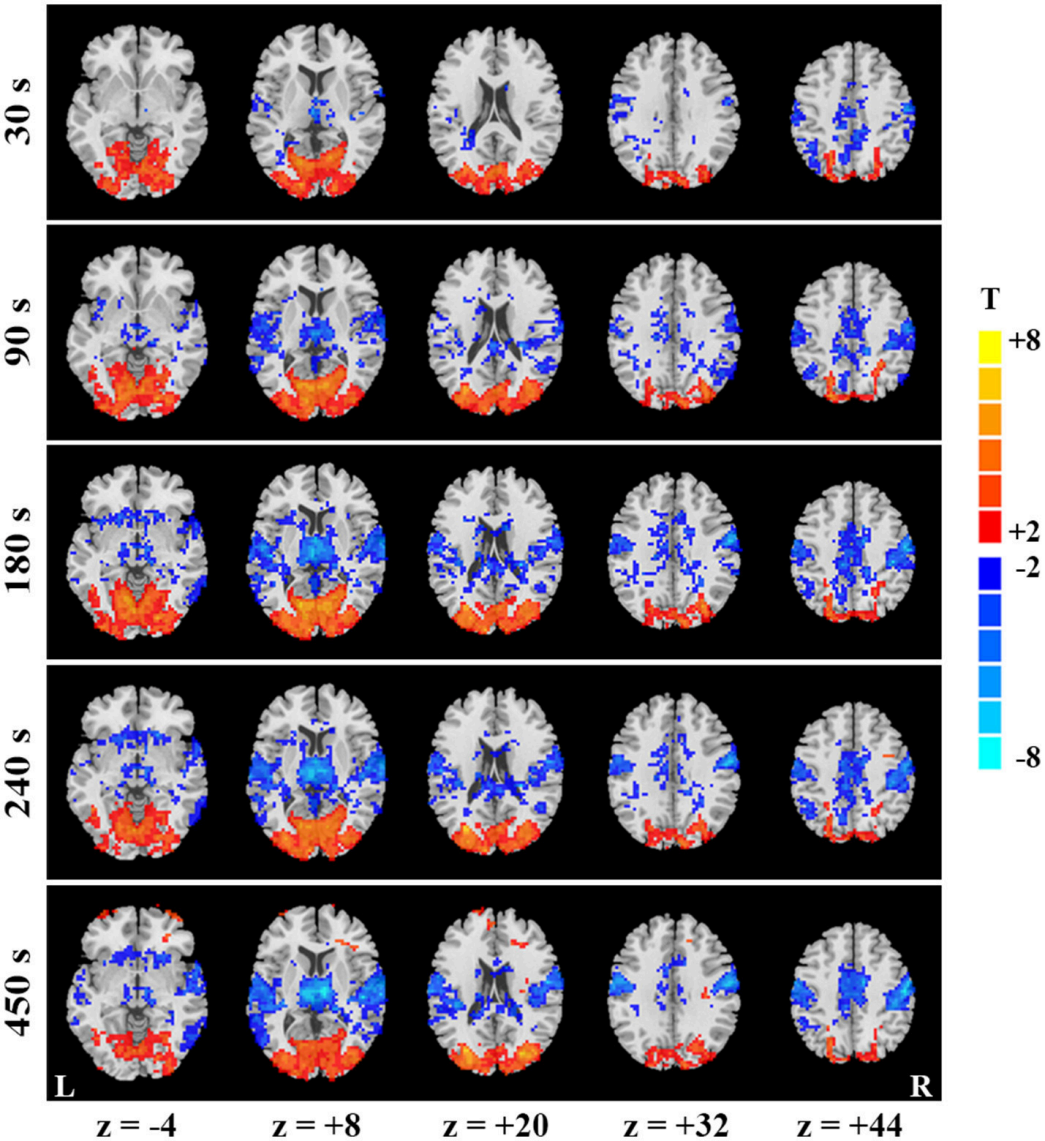
Temporal evolution of SD-based EO/EC differences for the continuous data. EO/EC SD differences based on the continuous data analyzed with GMN in different window (window length = 30, 90, 180, 240, and 450 s) were shown. The corrected P values were thresholded at *p* < 0.05. The warm colors indicate the regions with significantly increased SD in EO than EC, and the cool colors indicate the opposite (L, left hemisphere; R, right hemisphere).

To explain the differences between results of continuous and block-designed data, we further manifested the temporal dynamics of SD-based EO/EC differences for the continuous data. Specifically, at the beginning of scan (i.e., the first 30 s), some spatially discrete voxels showed decreased SD in PSMC and PAC for the data analyzed with GMN (*p* < 0.05, corrected, Figure 2, the top row). The decreases in PAC and PSMC were larger as the window lengths increased, and became stable until the window lengths reached 270 s for results with GMN (*p* < 0.05, corrected, Figure 2). Such trends were also found in the data analyzed without GMN (Supplementary Figure 2). The temporal changes of visual areas were not so obvious. Complete description for all windows could be seen in the videos in the Supplementary Materials.

**Figure 3:**
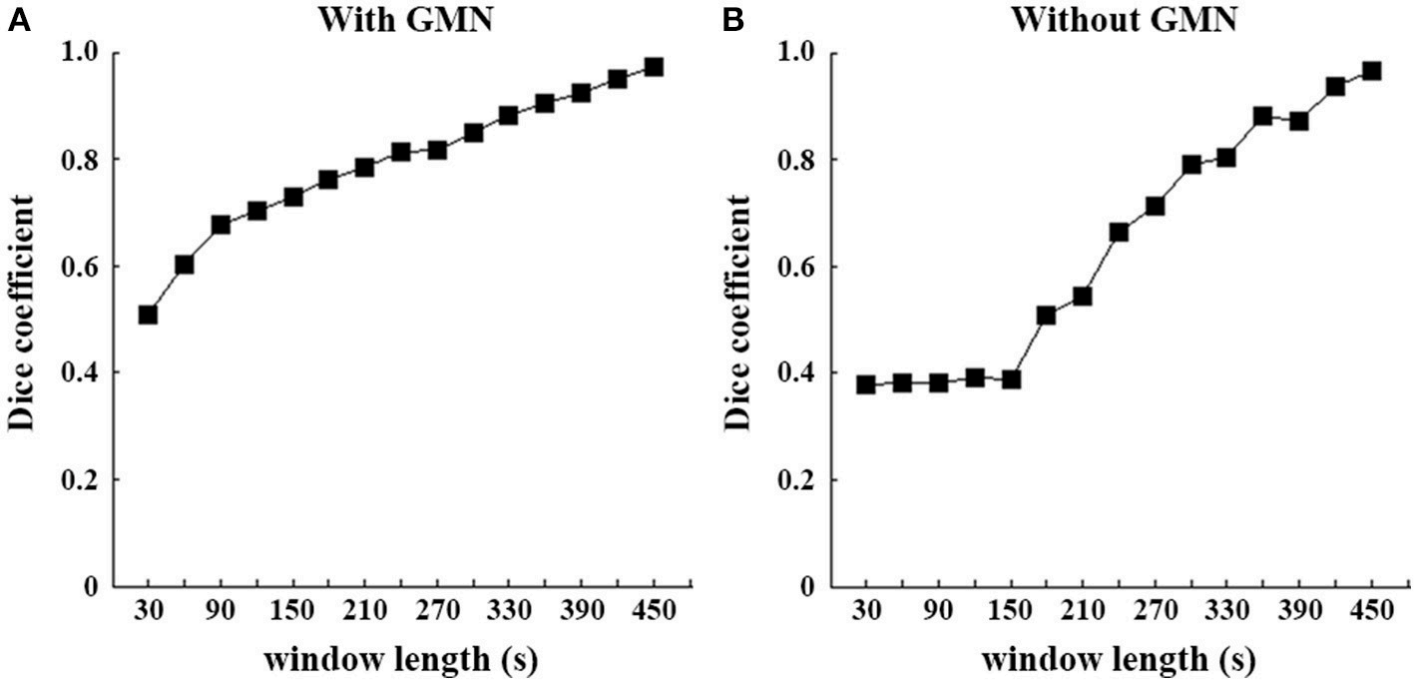
Spatial similarity of SD-based EO/EC differences between each window and the full-length data. Dice coefficients were computed between each temporal window and the full-length continuous data. The left **(A)** is the result based on the data with GMN and the right **(B)** is that without.

For the illustration purpose, we calculated the dice coefficients to reveal the similarity between patterns of each window and those of full length. The slow changes of SD with increasing window lengths were evident (Figure 3), irrespective of GMN.

## Discussion

In this study, we focused on the comparisons between BOLD derived variability- and mean-based EO/EC differences, within the same subjects and under the same experimental environment. Our findings demonstrated that the variability-based EO/EC differences are highly consistent with previous findings and have largely distinct spatial distribution with traditional activation. We confirmed that BOLD variability could provide orthogonal views of brain function with mean-based activations. Moreover, we also demonstrated variability-based modulation is a slower process than typically considered for activation, and thus block design is less optimal than continuous design in characterizing BOLD variability.

### Spatial distinction between mean- and SD-based results

In our results, the distinction between mean- and SD-based EO/EC differences was evident both for block and continuous designs. For the block-designed data, the mean-based EO/EC differences (i.e., typical activation revealed by GLM) was quite prominent in PVC and lateral thalamus. We did not found the widespread deactivation of bilateral PSMC as have detected in darkness (Marx et al., 2003). Likewise, Marx et al. (2003) did not detect the PVC activation. Although Jao et al. (2013) did not found the impact of light/darkness on variability-based indices, our results as well as previous finding (Marx et al., 2004) imply that room illuminance can significantly affect EO/EC traditional activation (i.e., signal-mean differences). Such differences between different experimental environments also highlighted the importance of controlling room illuminance level when performing the EO/EC studies.

SD analysis on the block-designed data showed largely distinct pattern with activation results. In particular, the spatial extent in PVC became much smaller in SD results than activation. However, lateral clusters in secondary visual cortex were more significant for SD results, and decreases of SD in medial thalamus and PAC emerged. Using block design, a few studies have demonstrated between-condition differences in signal variability have distinct spatial patterns from the traditional activation regions in memory tasks with high attentional demand (Protzner et al., 2013; Garrett et al., 2014; Guitart-Masip et al., 2015). Thus, our findings expand their conclusions to simple sensory task.

For continuous data, the between-condition differences in SD were more prominent than in block designed data. However, we did not found any significant differences in signal mean. The areas of SD decreases in PAC and PSMC became much larger than in block design. The pattern exhibited an almost symmetric distribution and was highly consistent with our previous findings (Yan et al., 2009; Liu et al., 2013; Yuan et al., 2014; Zou et al., 2015), and was also similar with the results without GMN, suggesting the robustness of variability changes between continuous EO and EC. Although there is discrepancy in visual cortex between our findings and other groups (McAvoy et al., 2008; Bianciardi et al., 2009; Jao et al., 2013). They found the decrease of variability in PVC whereas we detected the increased SD only in bilateral extrastriate cortex. This is probably caused by the different visual presentation in EO condition we used.

### Comparisons between design types

In addition to the distinction between mean- and SD-based results, we also observed considerable differences between results of block-designed and continuous data. This is true both for mean and variability indices. However, such differences may be caused by different reasons. For mean-based results, activation in PVC and thalamus was prominent in block-designed data whereas no significant differences were detected in continuous data, irrespective of whether intensity scaling was performed. Since Zou et al. (2015) has actually shown that mean level of CBF was increased in PVC in continuous EO than EC, here we believe that no differences in BOLD signal mean does not imply that there is no changes in mean level of neuronal activities. The underlying true effect may be confounded by different scaling factors inherent in different BOLD-fMRI sessions when signal mean of continuous data were compared. It is well known that raw intensity of BOLD signal could vary remarkably across different fMRI sessions. As such, researchers in task fMRI studies are only interested in relative signal change. In our continuous data, the scaling factors may differ across subjects and even across EO and EC states within the same subject. Although we have employed intensity scaling during preprocessing, it should be noted that while this manipulation can correct the gain effect, it could also lead to artificially negative results. By contrast, this problem is not so terrible in block-designed data since the gain effect was almost constant during the same session.

For SD results, the significant regions identified in block-designed data are almost subset of those in continuous data. The positive regions were similar between the two design types whereas the spatial extent of negative regions became much smaller for the block-designed data. To explain such distinction, we examined the temporal evolution of SD-based differences for the continuous data. The results showed that the significant areas (especially the negative regions) gradually became larger as the window lengths increased both for the data analyzed with and without GMN. Our findings suggest that changes of SD in PAC and PSMC is a rather slow process and thus cannot be fully captured within the short blocks. It should be noted that the distinction of SD results between continuous and block-designed data is not due to differences in sample lengths, since the length of concatenated block-designed data, on which SD was calculated, is more than 200 s whereas the decreases of SD in bilateral PSMC and PAC in continuous data were evident as soon as nearly 90 s after the scan onset. The slow dynamics of SD results are in line with recent findings that more than 5 min is necessary for functional connectivity (FC) metrics to reach stable (Whitlow et al., 2011; Birn et al., 2013; Gonzalez-Castillo et al., 2014; Tomasi D. G. et al., 2016). Although SD and FC characterize different aspects of signal fluctuation, a few groups have recently found temporal coupling between dynamical FC and dynamical variability (Tomasi D. et al., 2016; Fu et al., 2017), implying their similar neuronal origins.

Importantly, the slow dynamics indicated that block design [especially the design in which block length is less than 1 min, as did in some previous studies, (Garrett et al., 2013a, 2014; Protzner et al., 2013; Guitart-Masip et al., 2015)], is not ideal for BOLD variability analysis. By contrast, continuous design may be more appropriate in that it is more suited to capture the slow fluctuations. Therefore, we believe the SD results based on continuous design (at least for the EO/EC paradigm) is more closed to the true effect. However, the validity of continuous design has not been fully established. Since BOLD-fMRI signal has arbitrary units and SD is proportional to gain factor of raw BOLD signal, cautions should be taken for the variability analysis of continuous data. Although the high reproducibility between our results and previous papers (Yan et al., 2009; Liu et al., 2013; Yuan et al., 2014; Zou et al., 2015) suggest comparison of SD of BOLD signal for the continuous design is feasible, it actually remains unclear whether continuous design is suitable as well in other domains in cognitive neuroscience. That may depend on the particular questions to be answered. Methodological studies to evaluate and establish the validity of continuous design are needed in the future.

### Variability- and mean-based EO/EC differences might reflect two distinct response modes

Notably, one possible explanation for the distinction between signal variability and mean is that the two metrics are different in sensitivity to detect between-condition differences. However, we believe it is more likely that SD- and mean-based EO/EC differences might reflect two quite different aspects of human brain response. There are two reasons. First, as we have performed signal intensity scaling and block-mean correction, our findings thus suggest that the changes of BOLD variability is independent of mean signal level. Second, and more importantly, we found their distributions exhibited almost orthogonal patterns within sensory systems. Specifically, traditional EO/EC activation was mainly in PVC whereas SD-based EO/EC differences were in bilateral PAC and PSMC, as well as secondary visual cortex.

Compared with PVC which is response for direct processing of visual input, the cortical regions with SD changes (here include PAC, PSMC, and secondary visual cortex) might be involved in the visual processing in a more indirect way. The decreases of SD in PAC and PSMC during EO may reflect active or passive suppression of excitability in these non-visual modalities in order to facilitate the processing of incoming visual stimuli (Baier et al., 2006; Mozolic et al., 2008). This notion is in line with Raichle's hypothesis that the resting human brain is restless but at the “ready state” (Raichle et al., 2001), which could be modulated in response to environment demands (Raichle, 2010). The roles of secondary visual cortex may be more complicated. Studies have observed secondary visual cortex was deactivated in somatosensory task (Kawashima et al., 1995), in which the authors interpreted it as “closing the mind's eyes.” It has also been found to be deactivated (Baier et al., 2006) and showed decreased occipital-temporal FC (Pelland et al., 2017) in auditory tasks. More interestingly, the secondary visual cortex could be activated by high contrast visual stimuli in the macaque monkeys with PVC lesion, which might be associated with the unconscious visual processing (Schmid et al., 2010). For our findings, we speculated the higher variability in secondary visual cortex might reflect the modulation of visual awareness, which in turn could enhance the excitability of visual cortex. Future work is required to test this hypothesis.

The observation of decreased SD in non-visual modalities is quite similar with the phenomenon of cross-modal compensation which has been widely reported in the literature of blind (Stevens et al., 1996; Lessard et al., 1998; Röder et al., 1999; Van Boven et al., 2000; Goldreich and Kanics, 2003; Gougoux et al., 2004; Voss et al., 2004). Blind usually develop superior abilities than sighted people in non-visual tasks, such as auditory localization (Lessard et al., 1998; Röder et al., 1999; Voss et al., 2004), pitch discrimination (Gougoux et al., 2004) and tactile acuity (Stevens et al., 1996; Van Boven et al., 2000; Goldreich and Kanics, 2003). Higher task performance in non-visual tasks for blind might be caused by loss of sight, a situation partly similar to EC during which brain variability of PSMC and PAC becomes larger. So far, it remains unclear whether the biological basis of superior tactility and auditory abilities for blind could be, at least in part, accounted for by the brain variability. It would be interesting to examine their relationship in the future.

## Conclusions

By using BOLD-fMRI and comprehensive comparisons, we provided more solid evidences to confirm that BOLD variability could represent orthogonal views of brain function with conventional activation, thus highlighted the importance of variability analysis in task fMRI studies. Furthermore, it could exhibit rather slow dynamics that cannot be fully captured by typical block design. Its behavior significance requires further investigation.

## Author contributions

D-QL designed research. P-WZ and X-JQ performed research. P-WZ, X-JQ, S-FQ, X-BW, R-DW, Q-YL, S-YL, LC, and D-QL wrote the paper.

### Conflict of interest statement

The authors declare that the research was conducted in the absence of any commercial or financial relationships that could be construed as a potential conflict of interest.
