# Supplementary material for: Distinction Between Variability-Based Modulation and Mean-Based Activation Revealed by BOLD-fMRI and Eyes-Open/Eyes-Closed Contrast
Source: Front Neurosci. 2018 Jul 31;12:516. doi: 10.3389/fnins.2018.00516 (PMC6079296; doi:10.3389/fnins.2018.00516)
Supplement: Supplementary file 1 [file Data_Sheet_1.PDF]

Supplementary Materials

Distinction between Variability-based Modulation and Mean-based Activation Revealed by BOLD-fMRI and Eyes-open/Eyes-closed Contrast

Pei-Wen Zhang[1], Xiu-Juan Qu[1], Shu-Fang Qian[1], Xin-Bo Wang[1], Rui-Di Wang[1], Qiu-Yue, Li[1], Shi-Yu Liu[1], Dong-Qiang Liu[1*]

***Correspondence to:**
Dong-Qiang Liu, Ph.D
Email: charlesliu116@gmail.com

1 Supplementary Figures

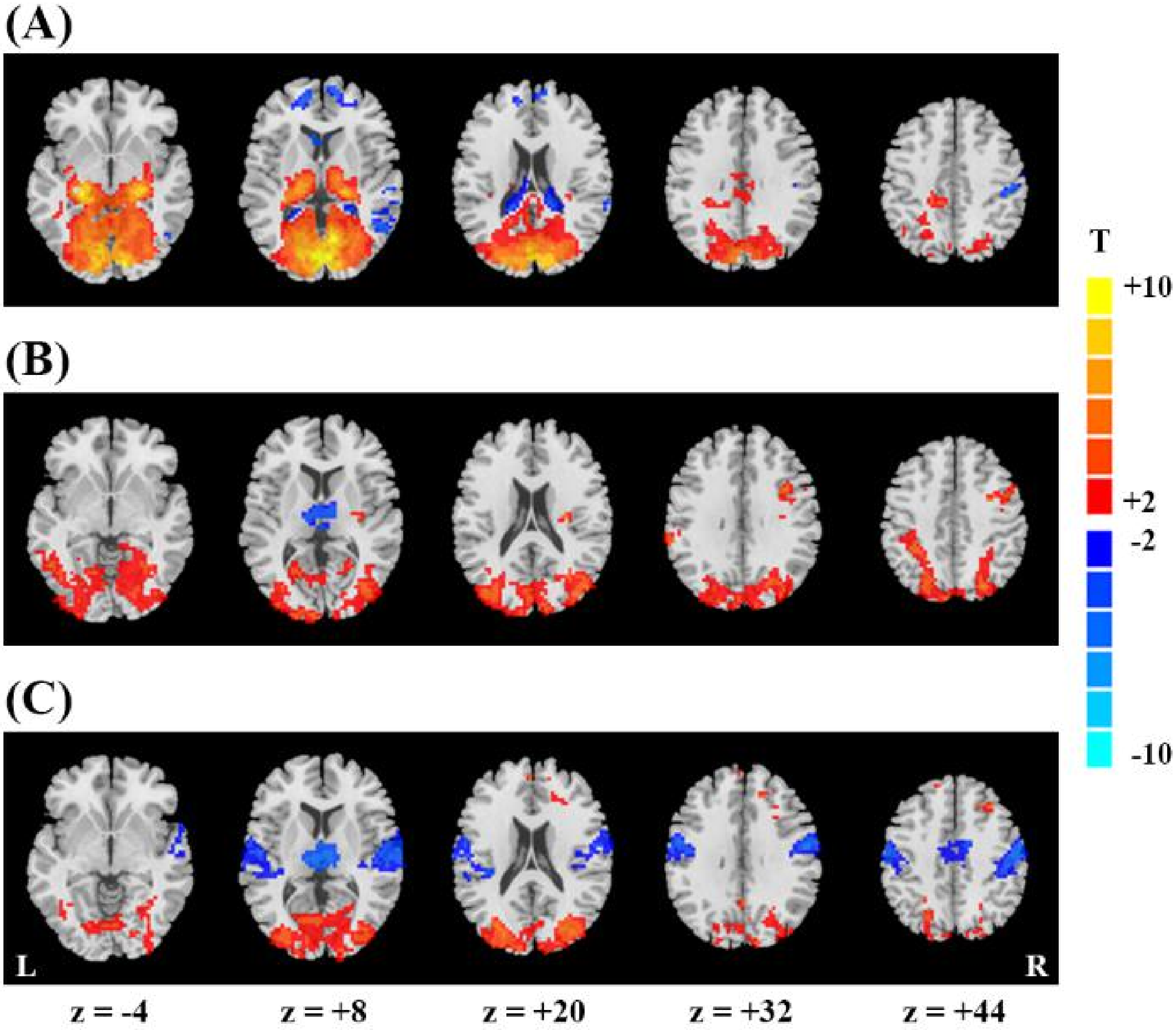

Supplementary Figure 1. Spatial maps of mean- and SD-based EO/EC differences. Activation

map **(A)**, and SD-based EO/EC differences for the block-designed **(B)** and continuous **(C)** data analyzed without GMN. The corrected P values were thresholded at $p < 0.05$. The warm colors indicate the regions with significantly increased activities in EO than EC, and the cool colors indicate the opposite (L = left hemisphere, R = right hemisphere).

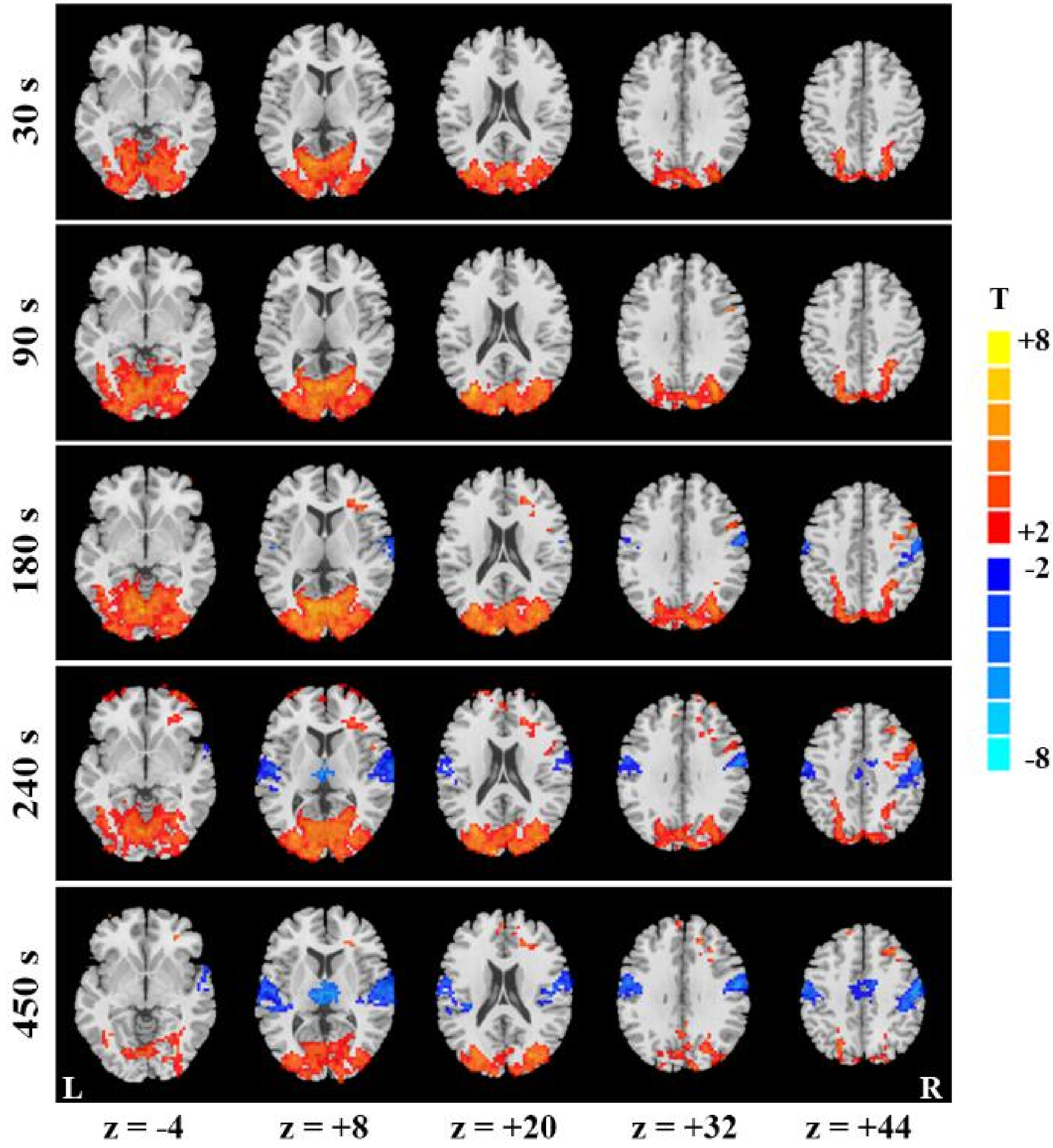

Supplementary Figure 2. Temporal evolution of SD-based EO/EC differences for the continuous data. EO/EC SD differences based on the continuous data analyzed without GMN in different windows (window length = 30 s, 90 s, 180 s, 240 s and 450 s) were shown. The corrected P values were thresholded at $p < 0.05$. The warm colors indicate the regions with significantly increased SD in EO than EC, and the cool colors indicate the opposite (L = left hemisphere, R = right

hemisphere).

2 Supplementary Video Legends

Supplementary Video 1. Temporal evolution of SD-based EO/EC differences for the continuous data with GMN. This video showed the results based on the continuous data analyzed with GMN in each of 15 windows (window length = 30 s, 60 s, …, 450 s). The corrected P values were thresholded at $p < 0.05$. The warm colors indicate the regions with significantly increased SD in EO than EC, and the cool colors indicate the opposite (L = left hemisphere, R = right hemisphere).

Supplementary Video 2. Temporal evolution of SD-based EO/EC differences for the continuous data without GMN. This video showed the corrected results based on the continuous data analyzed without GMN in each of 15 windows (window length = 30 s, 60 s, …, 450 s). The corrected P values were thresholded at $p < 0.05$. The warm colors indicate the regions with significantly increased SD in EO than EC, and the cool colors indicate the opposite (L = left hemisphere, R = right hemisphere).
